# The genome sequence of a scale worm, *Harmothoe impar *(Johnston, 1839)

**DOI:** 10.12688/wellcomeopenres.19570.1

**Published:** 2023-07-20

**Authors:** Patrick Adkins, Robert Mrowicki, Joanna Harley, Marine Biological Association Genome Acquisition Lab, Darwin Tree of Life Barcoding collective, Wellcome Sanger Institute Tree of Life programme, Wellcome Sanger Institute Scientific Operations: DNA Pipelines collective, Tree of Life Core Informatics collective, Darwin Tree of Life Consortium

**Affiliations:** 1 The Marine Biological Association, Plymouth, England, UK

**Keywords:** Harmothoe impar, (a scale worm), genome sequence, chromosomal, Phyllodocida

## Abstract

We present a genome assembly from an individual scale worm, *Harmothoe impar*; Annelida; Polychaeta; Phyllodocida; Polynoidae). The genome sequence is 1,512.3 megabases in span. Most of the assembly is scaffolded into 18 chromosomal pseudomolecules. The mitochondrial genome has also been assembled and is 15.37 kilobases in length.

## Species taxonomy

Eukaryota; Metazoa; Eumetazoa; Bilateria; Protostomia; Spiralia; Lophotrochozoa; Annelida; Polychaeta; Errantia; Phyllodocida; Polynoidae; *Harmothoe*; *Harmothoe impar* (Johnston, 1839) (NCBI:txid46595).

## Background

A scale worm found across the north-east Atlantic and into the Mediterranean, *Harmothoe impar* is often found under stones and inside kelp holdfasts, both intertidally and down to depths of 45 m. As with other polynoids, it is predatory, consuming small crustaceans and other small invertebrates, as well as being aggressive to the extent of cannibalisation to other members of the same species ( Bentley & Serries, 1992; Wolff, 1973). *H. impar* is found in the gut contents of several commercially important, mostly bottom-feeding fish species ( Pinnegar, 2014).

**Figure 1.**
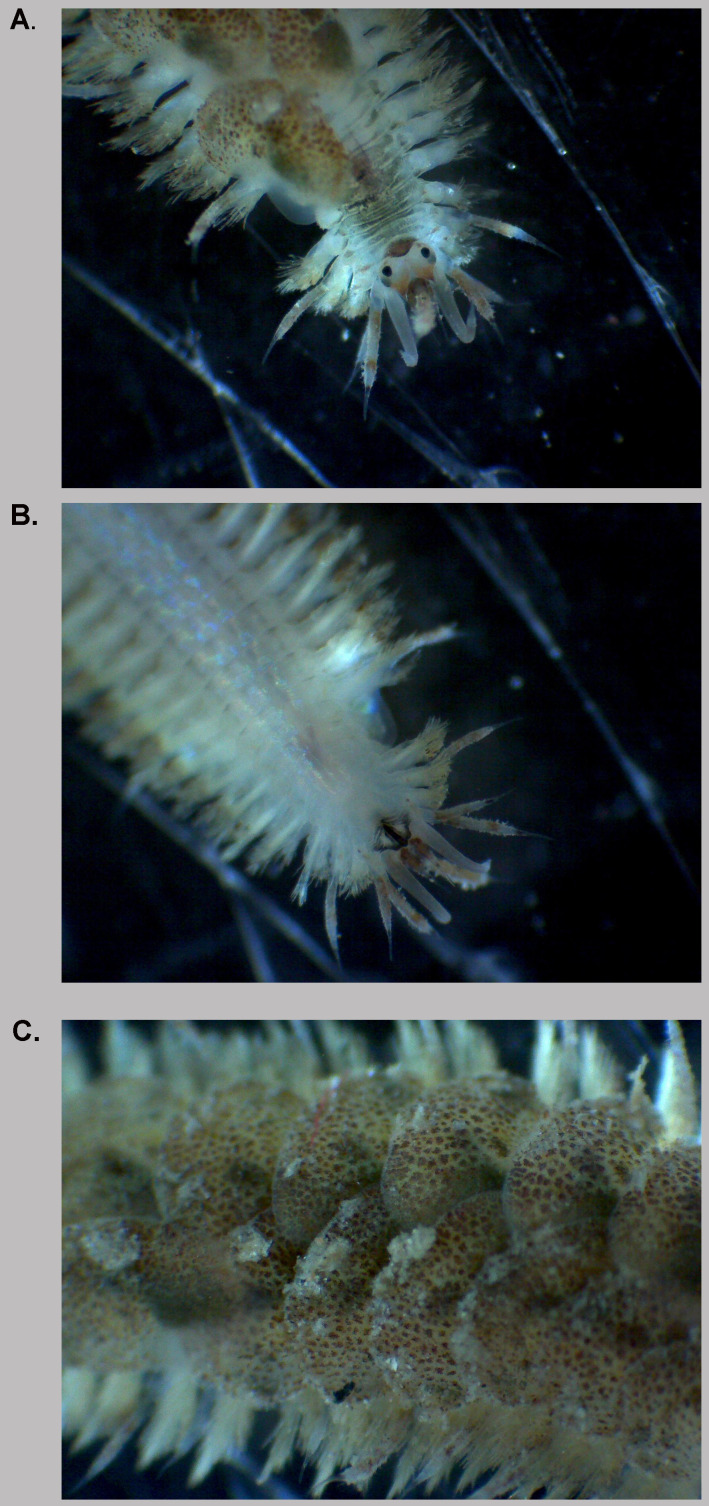
Photograph of the *Harmothoe impar* (wpHarImpa5) specimen used for genome sequencing. **A.** Head, dorsal view. **B.** Head, ventral view. **C.** Elytra.

Polynoids are typified by the presence of scales called elytra across their dorsum. *H. impar* is distinguished from other UK *Harmothoe* species primarily by these elytra, which are strongly spined and papillated, covering the worm dorsally almost in its entirety ( Figure 1C). The elytra have been shown in other members of the genus to be used by females to hold eggs, whilst ciliated tracks carry sperm towards them allowing direct fertilisation. The fertilised eggs are then brooded until released into the plankton as trochophores ( Daly, 1972). Bioluminescence has also been recorded across the family and members of the genus ( Livermore *et al.*, 2018; Moraes *et al.*, 2021), although it has yet to be described in this species.

The genome of *Harmothoe impar* was sequenced as part of the Darwin Tree of Life Project, a collaborative effort to sequence all named eukaryotic species in the Atlantic Archipelago of Britain and Ireland. This high-quality reference genome will provide the tools for further work with a variety of applications relating to this species. 

## Genome sequence report

The genome was sequenced from one *Harmothoe impar* specimen ( Figure 1) collected from Batten Bay South, Devon, UK (50.36, –4.13). A total of 30-fold coverage in Pacific Biosciences single-molecule HiFi long reads was generated. Primary assembly contigs were scaffolded with chromosome conformation Hi-C data. Manual assembly curation corrected 638 missing joins or mis-joins and removed 266 haplotypic duplications, reducing the assembly length by 9.71% and the scaffold number by 22.97%, and increasing the scaffold N50 by 12.34%.

**Figure 2.**
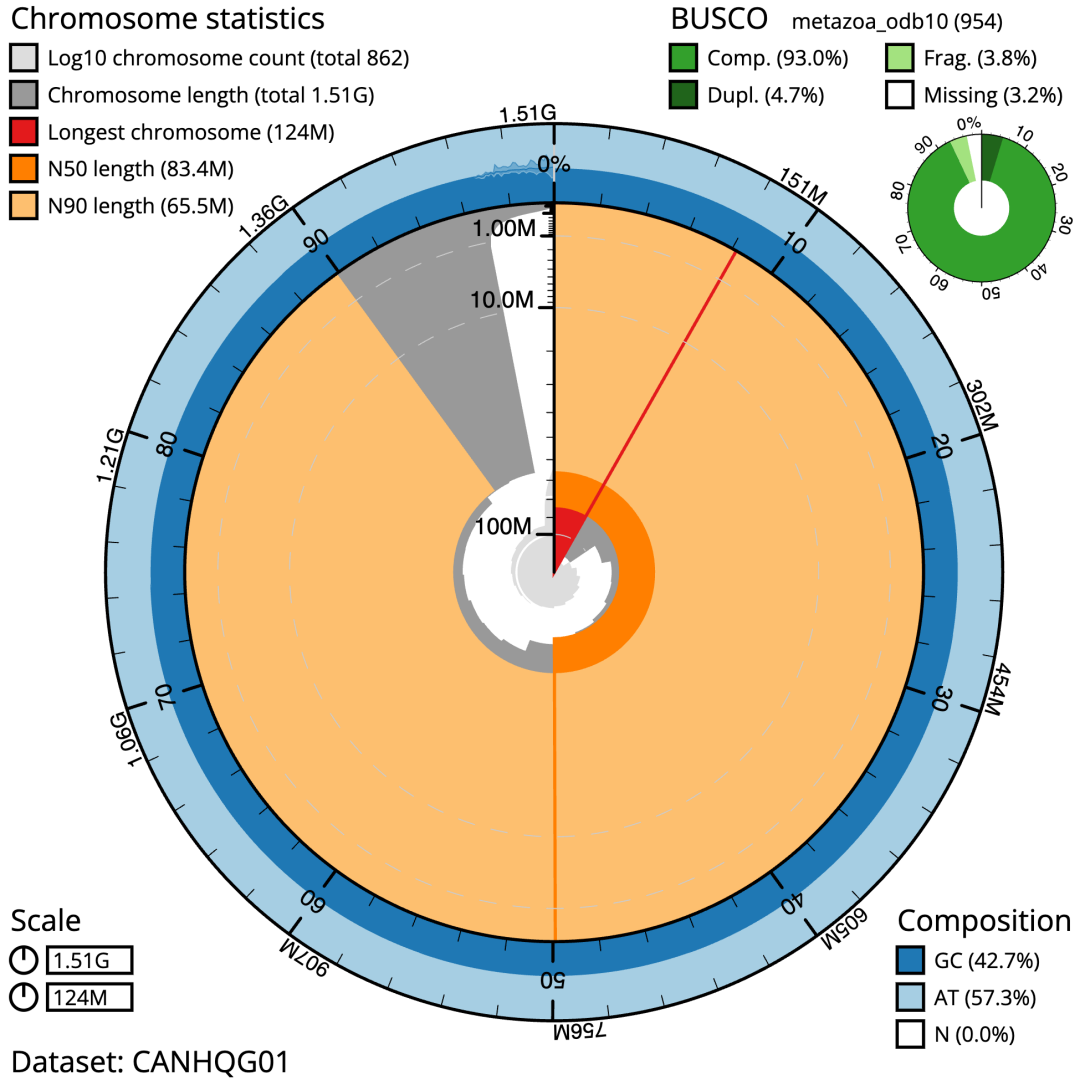
Genome assembly of *Harmothoe impar*, wpHarImpa5.1: metrics. The BlobToolKit Snailplot shows N50 metrics and BUSCO gene completeness. The main plot is divided into 1,000 size-ordered bins around the circumference with each bin representing 0.1% of the 1,512,319,410 bp assembly. The distribution of scaffold lengths is shown in dark grey with the plot radius scaled to the longest scaffold present in the assembly (124,100,948 bp, shown in red). . Orange and pale-orange arcs show the N50 and N90 scaffold lengths (83,397,641 and 65,536,652 bp), respectively. The pale grey spiral shows the cumulative scaffold count on a log scale with white scale lines showing successive orders of magnitude. The blue and pale-blue area around the outside of the plot shows the distribution of GC, AT and N percentages in the same bins as the inner plot. A summary of complete, fragmented, duplicated and missing BUSCO genes in the metazoa_odb10 set is shown in the top right. An interactive version of this figure is available at https://blobtoolkit.genomehubs.org/view/wpHarImpa5.1/dataset/CANHQG01/snail.

The final assembly has a total length of 1,512.3 Mb in 861 sequence scaffolds with a scaffold N50 of 83.4 Mb ( Table 1). Most (96.89%) of the assembly sequence was assigned to 18 chromosomal-level scaffolds. Chromosome-scale scaffolds confirmed by the Hi-C data are named in order of size ( Figure 2– Figure 5; Table 2). It appears that there are inversions between sister chromatids in the following approximate locations: Chromosome 1: 54–69 Mb, Chromosome 2: 14–80 Mb, Chromosome 12: 22–64 Mb, and Chromosome 14: 20–60 Mb While not fully phased, the assembly deposited is of one haplotype. Contigs corresponding to the second haplotype have also been deposited. The mitochondrial genome was also assembled and can be found as a contig within the multifasta file of the genome submission.

**Table 1.** Genome data for *Harmothoe impar*, wpHarImpa5.1.

Project accession data		
Assembly identifier	wpHarImpa5.1	
Species	*Harmothoe impar*	
Specimen	wpHarImpa5	
NCBI taxonomy ID	46595	
BioProject	PRJEB53239	
BioSample ID	SAMEA8419683	
Isolate information	wpHarImpa5 wpHarImpa3	
Assembly metrics *		*Benchmark*
Consensus quality (QV)	58.6	*≥ 50*
*k*-mer completeness	100%	*≥ 95%*
BUSCO **	C:93.0%[S:88.3%,D:4.7%], F:3.8%,M:3.2%,n:954	*C ≥ 95%*
Percentage of assembly mapped to chromosomes	96.89%	*≥ 95%*
Sex chromosomes	-	*localised homologous pairs*
Organelles	Mitochondrial genome assembled	*complete single alleles*
Raw data accessions		
PacificBiosciences SEQUEL II	ERR9836420–ERR9836422	
Hi-C Illumina	ERR9820267	
PolyA RNA-Seq Illumina	ERR10378012, ERR10378013	
Genome assembly		
Assembly accession	GCA_947462335.1	
*Accession of alternate haplotype*	GCA_947462385.1	
Span (Mb)	1,512.3	
Number of contigs	3,020	
Contig N50 length (Mb)	1.3	
Number of scaffolds	861	
Scaffold N50 length (Mb)	83.4	
Longest scaffold (Mb)	124.1	

* Assembly metric benchmarks are adapted from column VGP-2020 of “Table 1: Proposed standards and metrics for defining genome assembly quality” from ( Rhie *et al.*, 2021). ** BUSCO scores based on the metazoa_odb10 BUSCO set using v5.3.2. C = complete [S = single copy, D = duplicated], F = fragmented, M = missing, n = number of orthologues in comparison. A full set of BUSCO scores is available at https://blobtoolkit.genomehubs.org/view/wpHarImpa5.1/dataset/CANHQG01/busco.

**Figure 3.**
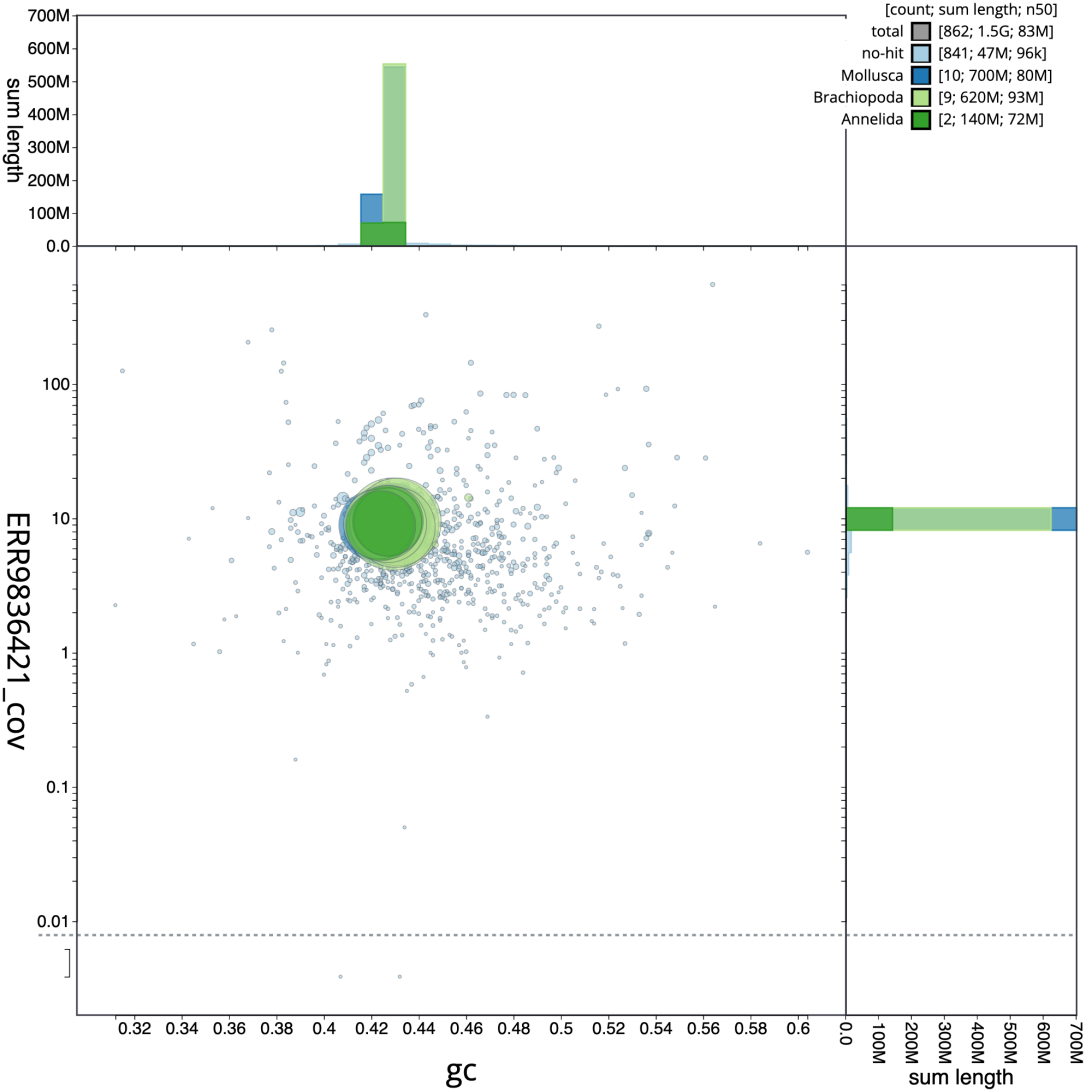
Genome assembly of *Harmothoe impar*, wpHarImpa5.1: BlobToolKit GC-coverage plot. Scaffolds are coloured by phylum. Circles are sized in proportion to scaffold length. Histograms show the distribution of scaffold length sum along each axis. An interactive version of this figure is available at https://blobtoolkit.genomehubs.org/view/wpHarImpa5.1/dataset/CANHQG01/blob.

**Figure 4.**
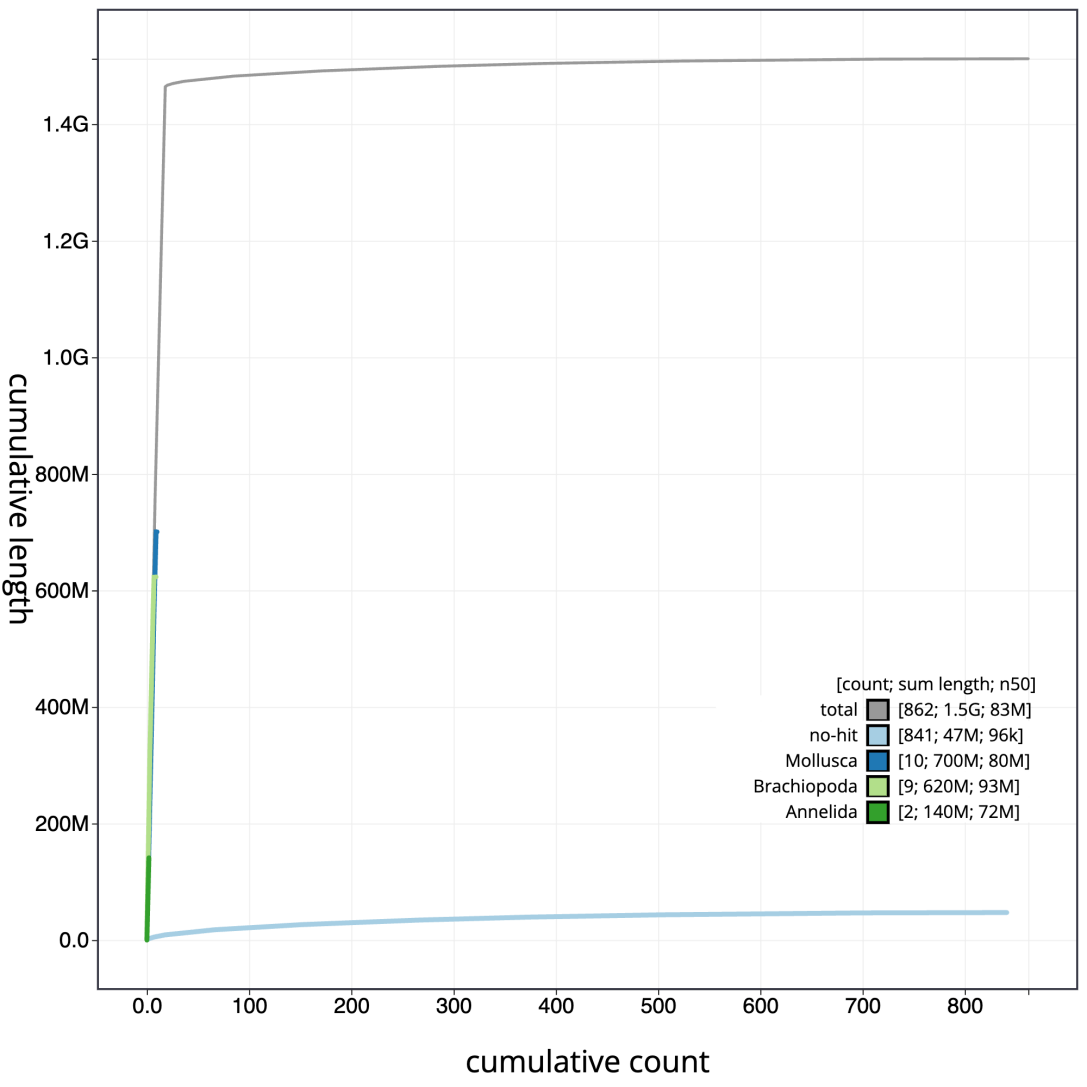
Genome assembly of *Harmothoe impar*, wpHarImpa5.1: BlobToolKit cumulative sequence plot. The grey line shows cumulative length for all scaffolds. Coloured lines show cumulative lengths of scaffolds assigned to each phylum using the buscogenes taxrule. An interactive version of this figure is available at https://blobtoolkit.genomehubs.org/view/wpHarImpa5.1/dataset/CANHQG01/cumulative.

**Figure 5.**
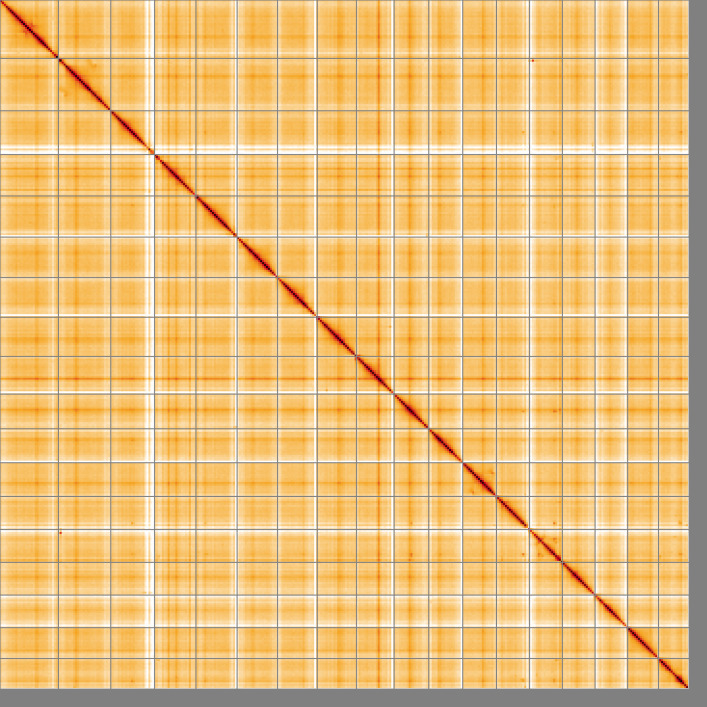
Genome assembly of *Harmothoe impar*, wpHarImpa5.1: Hi-C contact map of the wpHarImpa5.1 assembly, visualised using HiGlass. Chromosomes are shown in order of size from left to right and top to bottom. An interactive version of this figure may be viewed at https://genome-note-higlass.tol.sanger.ac.uk/l/?d=UxszSefoSW-pQuBgl6frig.

**Table 2.** Chromosomal pseudomolecules in the genome assembly of *Harmothoe impar*, wpHarImpa5.

INSDC accession	Chromosome	Length (Mb)	GC%
OX381704.1	1	124.1	43.0
OX381705.1	2	111.32	43.0
OX381706.1	3	93.07	43.0
OX381707.1	4	87.89	42.5
OX381708.1	5	87.48	43.0
OX381709.1	6	85.95	42.5
OX381710.1	7	84.46	43.0
OX381711.1	8	83.4	42.5
OX381712.1	9	79.9	43.0
OX381713.1	10	73.68	42.5
OX381714.1	11	72.18	43.0
OX381715.1	12	71.6	42.5
OX381716.1	13	70.4	43.0
OX381717.1	14	69.72	42.5
OX381718.1	15	69.57	42.5
OX381719.1	16	69.22	42.0
OX381720.1	17	65.54	42.5
OX381721.1	18	64.98	42.5
OX381722.1	MT	0.02	31.5

The estimated Quality Value (QV) of the final assembly is 58.6 with *k*-mer completeness of 100%, and the assembly has a BUSCO v5.3.2 completeness of 93.0% (single = 88.3%, duplicated = 4.7%), using the metazoa_odb10 reference set ( *n* = 954).

Metadata for specimens, spectral estimates, sequencing runs, contaminants and pre-curation assembly statistics can be found at https://links.tol.sanger.ac.uk/species/46595.

## Methods

### Sample acquisition and nucleic acid extraction

*Harmothoe impar* specimens were collected from Batten Bay South, Mount Batten, Devon, UK (latitude 50.36, longitude –4.13) on 2021-03-03. The specimens were taken by hand from underneath cobbles by Patrick Adkins and Rob Mrowicki (Marine Biological Association). Patrick Adkins and Joanna Harley (Marine Biological Association) identified the specimens, which were then preserved in liquid nitrogen.

DNA was extracted at the Tree of Life laboratory, Wellcome Sanger Institute (WSI). A sample of anterior body taken from specimen number wpHarImpa5 was weighed and dissected on dry ice with tissue set aside for Hi-C sequencing. Anterior body tissue was cryogenically disrupted to a fine powder using a Covaris cryoPREP Automated Dry Pulveriser, receiving multiple impacts. High molecular weight (HMW) DNA was extracted using the Qiagen MagAttract HMW DNA extraction kit. HMW DNA was sheared into an average fragment size of 12–20 kb in a Megaruptor 3 system with speed setting 30. Sheared DNA was purified by solid-phase reversible immobilisation using AMPure PB beads with a 1.8X ratio of beads to sample to remove the shorter fragments and concentrate the DNA sample. The concentration of the sheared and purified DNA was assessed using a Nanodrop spectrophotometer and Qubit Fluorometer and Qubit dsDNA High Sensitivity Assay kit. Fragment size distribution was evaluated by running the sample on the FemtoPulse system.

RNA was extracted from anterior body tissue of wpHarImpa6 in the Tree of Life Laboratory at the WSI using TRIzol, according to the manufacturer’s instructions. RNA was eluted in 50 μl RNAse-free water and its concentration assessed using a Nanodrop spectrophotometer and Qubit Fluorometer using the Qubit RNA Broad-Range (BR) Assay kit. Analysis of the integrity of the RNA was done using Agilent RNA 6000 Pico Kit and Eukaryotic Total RNA assay.

### Sequencing

Pacific Biosciences HiFi circular consensus DNA sequencing libraries were constructed according to the manufacturers’ instructions. Poly(A) RNA-Seq libraries were constructed using the NEB Ultra II RNA Library Prep kit. DNA and RNA sequencing were performed by the Scientific Operations core at the WSI on Pacific Biosciences SEQUEL II (HiFi) and Illumina NovaSeq 6000 (RNA-Seq) instruments. Hi-C data were also generated from specimen wpHarImpa3 using the Arimav2 kit and sequenced on the Illumina NovaSeq 6000 instrument.

### Genome assembly, curation and evaluation

Assembly was carried out with Hifiasm ( Cheng *et al.*, 2021) and haplotypic duplication was identified and removed with purge_dups ( Guan *et al.*, 2020). The assembly was then scaffolded with Hi-C data ( Rao *et al.*, 2014) using YaHS ( Zhou *et al.*, 2023). The assembly was checked for contamination and corrected using the gEVAL system ( Chow *et al.*, 2016) as described previously ( Howe *et al.*, 2021). Manual curation was performed using gEVAL, HiGlass ( Kerpedjiev *et al.*, 2018) and Pretext ( Harry, 2022). The mitochondrial genome was assembled using MitoHiFi ( Uliano-Silva *et al.*, 2022), which runs MitoFinder ( Allio *et al.*, 2020) or MITOS ( Bernt *et al.*, 2013) and uses these annotations to select the final mitochondrial contig and to ensure the general quality of the sequence.

A Hi-C map for the final assembly was produced using bwa-mem2 ( Vasimuddin *et al.*, 2019) in the Cooler file format ( Abdennur & Mirny, 2020). To assess the assembly metrics, the *k*-mer completeness and QV consensus quality values were calculated in Merqury ( Rhie *et al.*, 2020). This work was done using Nextflow ( Di Tommaso *et al.*, 2017) DSL2 pipelines “sanger-tol/readmapping” ( Surana *et al.*, 2023a) and “sanger-tol/genomenote” ( Surana *et al.*, 2023b). The genome was analysed within the BlobToolKit environment ( Challis *et al.*, 2020) and BUSCO scores ( Manni *et al.*, 2021; Simão *et al.*, 2015) were calculated.

Table 3 contains a list of relevant software tool versions and sources.

**Table 3.** Software tools: versions and sources.

Software tool	Version	Source
BlobToolKit	4.1.5	https://github.com/blobtoolkit/blobtoolkit
BUSCO	5.3.2	https://gitlab.com/ezlab/busco
gEVAL	N/A	https://geval.org.uk/
Hifiasm	0.16.1-r375	https://github.com/chhylp123/hifiasm
HiGlass	1.11.6	https://github.com/higlass/higlass
Merqury	MerquryFK	https://github.com/thegenemyers/MERQURY.FK
MitoHiFi	2	https://github.com/marcelauliano/MitoHiFi
PretextView	0.2	https://github.com/wtsi-hpag/PretextView
purge_dups	1.2.3	https://github.com/dfguan/purge_dups
sanger-tol/ genomenote	v1.0	https://github.com/sanger-tol/genomenote
sanger-tol/ readmapping	1.1.0	https://github.com/sanger-tol/readmapping/ tree/1.1.0
YaHS	yahs-1.1.91eebc2	https://github.com/c-zhou/yahs

### Legal and ethical review process for Darwin Tree of Life Partner submitted materials

The materials that have contributed to this genome note have been supplied by a Darwin Tree of Life Partner.

The submission of materials by a Darwin Tree of Life Partner is subject to the **‘Darwin Tree of Life Project Sampling Code of Practice’**, which can be found in full on the Darwin Tree of Life website here. By agreeing with and signing up to the Sampling Code of Practice, the Darwin Tree of Life Partner agrees they will meet the legal and ethical requirements and standards set out within this document in respect of all samples acquired for, and supplied to, the Darwin Tree of Life Project.

Further, the Wellcome Sanger Institute employs a process whereby due diligence is carried out proportionate to the nature of the materials themselves, and the circumstances under which they have been/are to be collected and provided for use. The purpose of this is to address and mitigate any potential legal and/or ethical implications of receipt and use of the materials as part of the research project, and to ensure that in doing so we align with best practice wherever possible.

The overarching areas of consideration are:

- Ethical review of provenance and sourcing of the material
- Legality of collection, transfer and use (national and international)

Each transfer of samples is further undertaken according to a Research Collaboration Agreement or Material Transfer Agreement entered into by the Darwin Tree of Life Partner, Genome Research Limited (operating as the Wellcome Sanger Institute), and in some circumstances other Darwin Tree of Life collaborators.

## Data Availability

European Nucleotide Archive: *Harmothoe impar* (a scale worm). Accession number PRJEB53239; https://identifiers.org/ena.embl/PRJEB53239. ( Wellcome Sanger Institute, 2022) The genome sequence is released openly for reuse. The *Harmothoe impar* genome sequencing initiative is part of the Darwin Tree of Life (DToL) project. All raw sequence data and the assembly have been deposited in INSDC databases. The genome will be annotated using available RNA-Seq data and presented through the Ensembl pipeline at the European Bioinformatics Institute. Raw data and assembly accession identifiers are reported in Table 1.

## Author information

Members of the Marine Biological Association Genome Acquisition Lab are listed here:

https://doi.org/10.5281/zenodo.4783604.

Members of the Darwin Tree of Life Barcoding collective are listed here: https://doi.org/10.5281/zenodo.4893703.

Members of the Wellcome Sanger Institute Tree of Life programme are listed here: https://doi.org/10.5281/zenodo.4783585.

Members of Wellcome Sanger Institute Scientific Operations: DNA Pipelines collective are listed here: https://doi.org/10.5281/zenodo.4790455.

Members of the Tree of Life Core Informatics collective are listed here: https://doi.org/10.5281/zenodo.5013541.

Members of the Darwin Tree of Life Consortium are listed here: https://doi.org/10.5281/zenodo.4783558.
