# Supplementary material for: The genome sequence of a scale worm, Harmothoe impar (Johnston, 1839)
Source: Wellcome Open Res. 2023 Jul 20;8:315. [Version 1] doi: 10.12688/wellcomeopenres.19570.1 (PMC10541532; doi:10.12688/wellcomeopenres.19570.1)
Supplement: Peer review history

# Reviewer report: Reviewer response for version 1

**DOI:** 10.21956/wellcomeopenres.21680.r67731

**Published:** 2023-09-29

**Authors:** José María Martín-Durán

**Affiliations:** 1 School of Biological and Behavioural Sciences, Queen Mary University of London, London, UK

This genome note reports the assembly and annotation of the genome of the polynoid annelid worm Harmothoe impar, a typical dweller of the UK coasts. Within annelids, the Errantia clade is under-represented in genomic efforts. Therefore, the sequencing of a polynoid will benefit the study and conservation of this central member of the marine macro-fauna of the UK, as well as evolutionary and comparative genomic analyses. The methods are gold-standard, and the reported assembly is consistent with the high-quality datasets produced by the DToL.

Are sufficient details of methods and materials provided to allow replication by others?

Yes

Is the rationale for creating the dataset(s) clearly described?

Yes

Are the datasets clearly presented in a useable and accessible format?

Yes

Are the protocols appropriate and is the work technically sound?

Yes

Reviewer Expertise:

EvoDevo, comparative genomics, annelids

I confirm that I have read this submission and believe that I have an appropriate level of expertise to confirm that it is of an acceptable scientific standard.

# Reviewer report: Reviewer response for version 1

**DOI:** 10.21956/wellcomeopenres.21680.r63704

**Published:** 2023-08-17

**Authors:** Stéphane Hourdez

**Affiliations:** 1 Laboratoire d’Ecogéochimie des Environnements Benthiques, Observatoire Océanologique de Banyuls, Sorbonne Université, Banyuls-sur-Mer, France

This data paper reports the sequencing and assembly of the first species of Polynoidae (Annelida, Polychaeta) available to date (out of ~1000 described species worldwide). It belongs to a typically shallow-water genus of the family, which comprises ~150 species. In European waters, over 50 species are known in the family. Other closely-related families are present and are also considered for sequencing by DTOL initiative. 

 This resource has already been used by a research team which studied adaptation in a deep-sea hydrothermal species from the Indian Ocean (He et al. 2023).

Are sufficient details of methods and materials provided to allow replication by others?

Yes

Is the rationale for creating the dataset(s) clearly described?

Yes

Are the datasets clearly presented in a useable and accessible format?

Yes

Are the protocols appropriate and is the work technically sound?

Yes

Reviewer Expertise:

Evolution of adaptations to extreme environments, taxonomy of annelids.

I confirm that I have read this submission and believe that I have an appropriate level of expertise to confirm that it is of an acceptable scientific standard.

## References

[1] : Genomic Analysis of a Scale Worm Provides Insights into Its Adaptation to Deep-Sea Hydrothermal Vents. *Genome Biol Evol* .2023;15(7) : 10.1093/gbe/evad125 10.1093/gbe/evad125 37401460PMC10348049
